# Ultrasound activates mechanosensitive TRAAK K^+^ channels through the lipid membrane

**DOI:** 10.1073/pnas.2006980118

**Published:** 2021-02-04

**Authors:** Ben Sorum, Robert A. Rietmeijer, Karthika Gopakumar, Hillel Adesnik, Stephen G. Brohawn

**Affiliations:** ^a^Department of Molecular and Cell Biology, University of California, Berkeley, CA 94720;; ^b^Helen Wills Neuroscience Institute, University of California, Berkeley, CA 94720;; ^c^Biophysics Graduate Program, University of California, Berkeley, CA 94720

**Keywords:** mechanosensation, ultrasound, K2P ion channels, neuromodulation, sonogenetics

## Abstract

Ultrasound modulates the electrical activity of excitable cells and offers advantages over other neuromodulatory techniques; for example, it can be noninvasively transmitted through the skull and focused to deep brain regions. However, the fundamental cellular, molecular, and mechanistic bases of ultrasonic neuromodulation are largely unknown. Here, we demonstrate ultrasound activation of the mechanosensitive K^+^ channel TRAAK with submillisecond kinetics to an extent comparable to canonical mechanical activation. Single-channel recordings reveal a common basis for ultrasonic and mechanical activation with stimulus-graded destabilization of long-duration closures and promotion of full conductance openings. Ultrasonic energy is transduced to TRAAK through the membrane in the absence of other cellular components, likely increasing membrane tension to promote channel opening. We further demonstrate ultrasonic modulation of neuronally expressed TRAAK. These results suggest mechanosensitive channels underlie physiological responses to ultrasound and could serve as sonogenetic actuators for acoustic neuromodulation of genetically targeted cells.

## Significance

Ultrasound stimulation modulates the electrical activity of excitable cells, including in neurons of the brain and central nervous system. Compared to other neuromodulatory techniques, ultrasound offers several advantages; for example, it can be noninvasively transmitted through the skull and focused to deep brain regions. However, the molecular basis underlying the effects of ultrasound on neural activity is not known. Here, we show that ultrasound activates the mechanosensitive ion channel TRAAK through the membrane in a manner analogous to canonical mechanical activation, likely by increasing membrane tension to promote channel opening. These results suggest mechanosensitive channels underlie physiological responses to ultrasound and could serve as tools for acoustic neuromodulation of genetically targeted cells.

Manipulating cellular electrical activity is central to basic research and is clinically important for the treatment of neurological disorders including Parkinson’s disease, depression, epilepsy, and schizophrenia (1–4). Optogenetics, chemogenetics, deep brain stimulation (DBS), transcranial electrical stimulation, and transcranial magnetic stimulation are widely utilized neuromodulatory techniques, but each is associated with physical or biological limitations (5). Transcranial stimulation affords poor spatial resolution; deep brain stimulation and optogenetic manipulation typically require surgical implantation of stimulus delivery systems, and optogenetic and chemogenetic approaches necessitate genetic targeting of light- or small-molecule–responsive proteins.

Ultrasound was first recognized to modulate cellular electrical activity almost a century ago, and ultrasonic neuromodulation has since been widely reported in the brain, peripheral nervous system, and heart of humans and model organisms (5–12). Ultrasonic neuromodulation has garnered increased attention for its advantageous physical properties. Ultrasound penetrates deeply through biological tissues and can be focused to sub-mm (3) volumes without transferring substantial energy to overlaying tissue, so it can be delivered noninvasively, for example, to deep structures in the brain through the skull. Notably, ultrasound generates excitatory and/or inhibitory effects depending on the system under study and stimulus paradigm (5, 13, 14).

The mechanisms underlying the effects of ultrasound on excitable cells remain largely unknown (5, 13). Ultrasound can generate a combination of thermal and mechanical effects on targeted tissue (15, 16) in addition to potential off-target effects through the auditory system (17, 18). Thermal and cavitation effects, while productively harnessed to ablate tissue or transiently open the blood–brain barrier (19), require stimulation of higher power, frequency, and/or duration than typically utilized for neuromodulation (5). Intramembrane cavitation or compressive and expansive effects on lipid bilayers could generate nonselective currents that alter cellular electrical activity (5, 13). Alternatively, ultrasound could activate mechanosensitive ion channels through the deposition of acoustic radiation force that increases membrane tension or geometrically deforms the lipid bilayer (5, 15). Consistent with this notion, behavioral responses to ultrasound in *Caenorhabditis elegans* require mechanosensitive, but not thermosensitive, ion channels (20), and a number of mechanosensitive (and force-sensitive, but noncanonically mechanosensitive) ion channels have been implicated in cellular responses to ultrasound including two-pore domain K^+^ channels (K2Ps), Piezo1, MEC-4, TRPA1, MscL, and voltage-gated Na^+^ and Ca^2+^ channels (20–24, 25). Precisely how ultrasound impacts the activity of these channels is not known.

To better understand mechanisms underlying ultrasonic neuromodulation, we investigated the effects of ultrasound on the mechanosensitive ion channel TRAAK (26, 27). K2P channels including TRAAK are responsible for so called “leak-type” currents because they approximate voltage- and time-independent K^+^-selective holes in the membrane, although more complex gating and regulation of K2P channels is increasingly appreciated (28, 29). TRAAK has a very low open probability in the absence of membrane tension and is robustly activated by force through the lipid bilayer (30–32). Mechanical activation of TRAAK involves conformational changes that prevent lipids from entering the channel to block K^+^ conduction (31). Gating conformational changes are associated with shape changes that expand the channel and make it more cylindrical in the membrane plane upon opening. These shape changes are energetically favored in the presence of membrane tension, resulting in a tension-dependent energy difference between states that favors channel opening (31). TRAAK is expressed in neurons and has been localized exclusively to nodes of Ranvier, the excitable action potential propagating regions of myelinated axons (33, 34). TRAAK is found in most (∼80%) myelinated nerve fibers in both the central and peripheral nervous systems, where it accounts for ∼25% of basal nodal K^+^ currents. As in heterologous systems, mechanical stimulation robustly activates nodal TRAAK. TRAAK is functionally important for setting the resting potential and maintaining voltage-gated Na^+^ channel availability for spiking in nodes; loss of TRAAK function impairs high-speed and high-frequency nerve conduction (33, 34). Changes in TRAAK activity therefore appear well poised to widely impact neuronal excitability.

We find that low-intensity and short-duration ultrasound rapidly and robustly activates TRAAK channels. Activation is observed in patches from TRAAK-expressing *Xenopus* oocytes, in patches containing purified channels reconstituted into lipid membranes, and in TRAAK-expressing mouse cortical neurons. Single-channel recordings reveal that canonical mechanical and ultrasonic activation are accomplished through a shared mechanism. We conclude that ultrasound activates TRAAK through the lipid membrane, likely by increasing membrane tension to promote channel opening. This work demonstrates direct mechanical activation of an ion channel by ultrasound using purified and reconstituted components, is consistent with endogenous mechanosensitive channel activity underlying physiological effects of ultrasound, and provides a framework for the development of exogenously expressed sonogenetic tools for ultrasonic control of neural activity.

## Results

We used a recording setup schematized in *SI Appendix*, Fig. S1*A* to isolate mechanical effects of ultrasound mediated through the membrane on ion channels. An ultrasound transducer is connected through tubing to a hole in the recording chamber filled with bath solution. Patched membranes are positioned directly above the transducer face at the position of maximum ultrasonic intensity to eliminate impedance differences between the transducer and membrane and associated surface effects. We designed stimulation protocols to minimize bath temperature increases (to less than ∼0.05 °C, unless otherwise noted, *SI Appendix*, Fig. S2*B*).

**Fig. 1.**
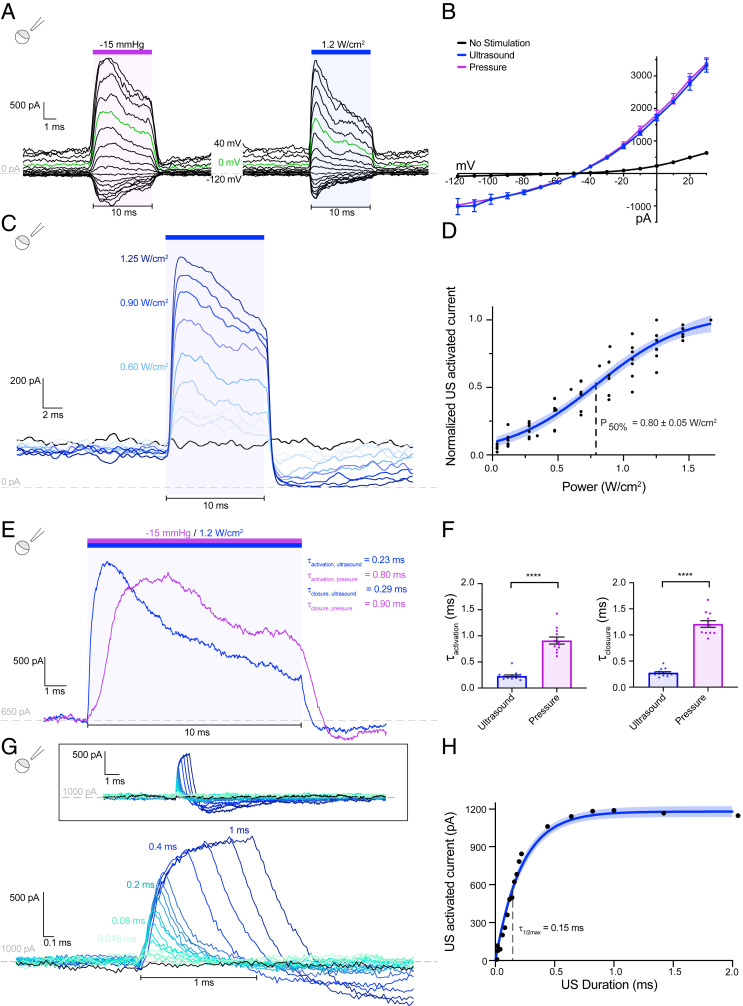
Ultrasound activates macroscopic TRAAK currents in *Xenopus* oocyte patches. (*A*) The currents recorded from an inside-out patch of a TRAAK-expressing oocyte during a voltage-step protocol (V_hold_ = −50 mV, V_test_ = −120 to +40 mV, ΔV = 10 mV). A pressure (−15 mmHg, purple bar, *Left*) or ultrasound step (1.2 W/cm^2^ at 5 MHz, blue bar, *Right*) was applied during each voltage step. A dashed line indicates 0 current, and a green trace corresponds to V_test_ = 0 mV. (*B*) The current–voltage relationship of data in *A*. The average current before stimulation (black), peak currents during pressure (purple), and ultrasound (blue) stimulation are shown (mean ± SEM, *n* = 3 sweeps). (*C*) An overlay of currents during steps of increasing ultrasound power (V_test_ = 0 mV). (*D*) The normalized ultrasound-induced TRAAK current versus ultrasound power (V_test_ = 0 mV). A Boltzmann fit with 95% CI is shown (*n* = 6 patches from 3 cells). (*E*) An overlay of the TRAAK current from the same patch in response to ultrasound (blue) and pressure (purple) (V_test_ = 0 mV). (*F*) The time constant of channel activation (left) and closure (right) in response to ultrasound (blue) and pressure (purple); 𝜏_activation,_ _ultrasound_ = 0.23 ± 0.02 ms, 𝜏_activation,_ _pressure_ = 0.91 ± 0.07 ms, 𝜏_close,_ _ultrasound_ = 0.28 ± 0.02, and 𝜏_close,_ _pressure_ = 1.21 ± 0.06 (*n* = 15 and 12 records for ultrasound and pressure, respectively, from 3 patches from 3 cells), ****P < 0.0001, Welch’s *t* test. (*G*) An overlay of TRAAK response to ultrasonic stimulation of increasing duration, colored from green to blue (V_test_ = 0 mV). (*H*) The maximum current response versus stimulus duration (V_hold_ = 0 mV). A fit with 95% CI is shown.

We first asked whether ultrasound activates TRAAK channels expressed in cells. As expected, pressure stimulation robustly increased TRAAK currents in patches excised from *Xenopus* oocytes (Fig. 1 *A* and *B*) (27, 32). We note that it is not pressure, per se, that directly activates TRAAK. Rather, pressure application increases membrane tension in the patch to promote channel opening. Strikingly, brief pulses of low intensity ultrasound (10 ms, 5 MHz, 1.2 W/cm^2^) similarly increased TRAAK currents. Like basal and pressure-stimulated TRAAK currents, ultrasound-stimulated currents were K^+^ selective with a reversal potential near the Nernst equilibrium potential for K^+^ (E_K+_ = −59 mV) (Fig. 1 *A* and *B*). Consistent with previous reports, the degree of outward rectification decreased as channel activity increased (35). Increasing steps of ultrasound power increasingly activated TRAAK current (Fig. 1 *C* and *D*) with a midpoint power of 0.80 ± 0.05 W/cm^2^ and 10 to 90% activation occurring between 0.3 and 1.1 W/cm^2^_._ At the highest ultrasound intensities tested, TRAAK was activated 21.9 ± 5.2-fold (mean ± SEM, *n* = 6). Ultrasound did not activate a related, but nonmechanosensitive, K2P ion channel TASK2 (*SI Appendix*, Fig. S1 *C* and *D*).

Ultrasonic and pressure stimulation both result in a rapid increase in current that decays while stimulus is maintained (Fig. 1 *E* and *F*). Similar desensitization of TRAAK following mechanical activation has been described (36). Ultrasound activates TRAAK currents approximately four times faster than pressure (𝜏_activation,_ _ultrasound_ = 0.23 ± 0.02 ms, 𝜏_activation,_ _pressure_ = 0.91 ± 0.07 ms (mean ± SEM, *n* = 15 and 12 records, respectively, from 3 patches from 3 cells) (Fig. 1*F*). Ultrasound-activated currents similarly decay faster than pressure-activated currents upon stimulus removal (𝜏_close,_ _ultrasound_ = 0.28 ± 0.02, 𝜏_close,_ _pressure_ = 1.21 ± 0.06 (*n* = 15 and 12 records, respectively, from 3 patches from 3 cells). The difference in macroscopic kinetics is at least partially explained by differences in the time required to deliver each stimulus. In our setup, negative pressure directed toward the patch pipette increased with a time constant of 1.3 ms, while ultrasound rise time is orders of magnitude faster. Therefore, the measured ultrasound activation kinetics more accurately represent intrinsic TRAAK kinetics, while those measured following pressure stimulations are filtered by the pressure clamp device. A consequence of the rapid ultrasonic activation of TRAAK is that even brief stimulation can activate large currents (Fig. 1 *G* and *H*); 0.15-ms and 0.8-ms stimulation result in ∼50% and ∼95% maximal TRAAK current, respectively.

We note that currents following termination of ultrasound stimulation are typically lower than the basal current for several milliseconds before returning to their baseline level (Fig. 1 *A*, *E*, and *G*). This is similarly observed following pressure stimulation (Fig. 1 *A* and *E*). In the case of pressure, this has been attributed to recruitment of an additional lipid bilayer into the patch during stretching, which, upon stimulus removal, results in transiently lower basal tension and reduced channel open probability (32, 36). The similar effect observed following ultrasound and pressure stimulation is consistent with both stimuli generating membrane tension that opens TRAAK channels.

**Fig. 2.**
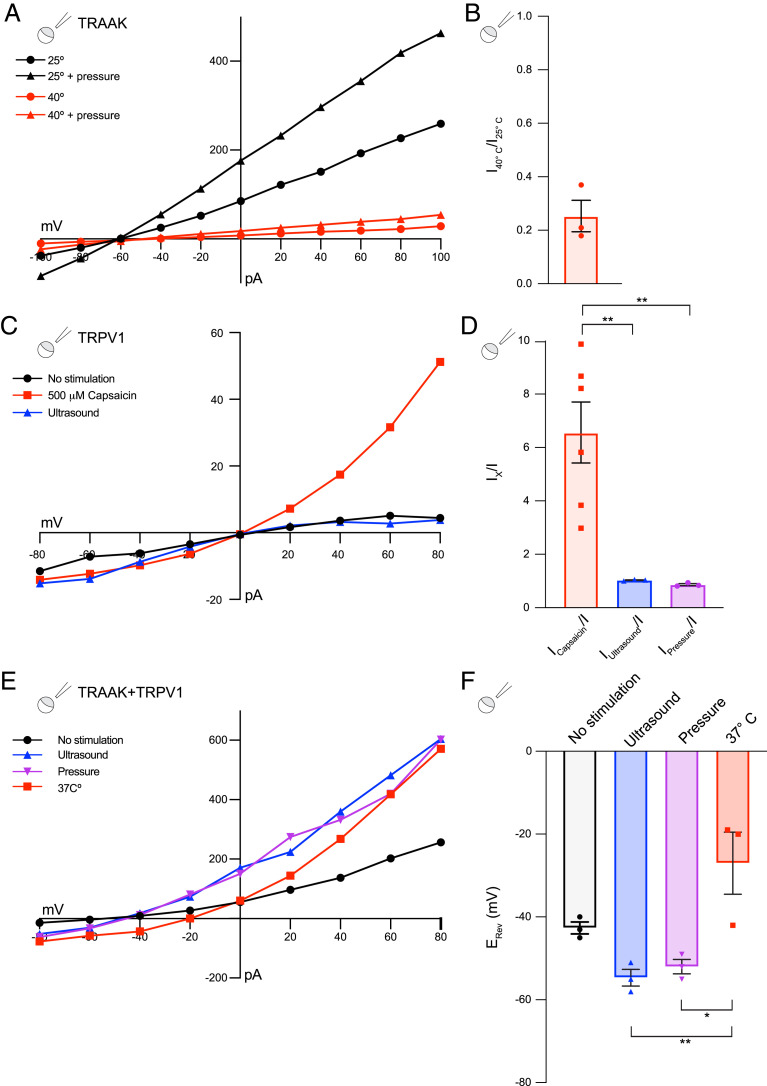
Mechanosensitive TRAAK channels, but not thermosensitive TRPV1 channels, are activated by ultrasound. (*A*) A representative current–voltage relationship recorded from an inside-out patch of a TRAAK-expressing oocyte without (circles) and with applied pressure (triangles) at 25 °C (black) and 40 °C (red). (*B*) The reduction of the TRAAK current at 40 °C relative to 25 °C recorded at 0 mV (relative activity 0.25 ± 0.06 [mean ± SEM, *n* = 3 patches]). (*C*) A representative current–voltage relationship recorded from an inside-out patch of a TRPV1-expressing oocyte without stimulation (black circles), during ultrasound stimulation (10 ms, 0.6 W/cm^2^ at 5 MHz, blue triangles), and following application of 500 µM capsaicin (red squares). (*D*) The fold activation of TRPV1 currents at +100 mV during the application of 500 µM capsaicin (red), during ultrasound stimulation (10 ms, 0.6 W/cm^2^ at 5 MHz blue), or during negative pressure stimulation (10 ms, −35 mmHg purple) (6.5 ± 1.1, 1.0 ± 0.01 and 0.9 ± 0.03-fold, mean ± SEM, *n* = 6, 3, and 3 patches, respectively), **P < 0.01, one-way ANOVA with Tukey correction). (*E*) A representative current–voltage relationship recorded from an inside-out patch of a TRAAK- and TRPV1-coexpressing oocyte without stimulation (black circles), during ultrasound stimulation (10 ms, 0.5 W/cm^2^ at 5 MHz, blue triangles), during pressure application (10 ms, −35 mmHg, purple inverted triangles), or during 37 °C bath solution application (red squares). (*F*) The reversal potential of currents recorded from TRAAK- and TRPV1-coexpressing oocyte patches under different conditions colored as in *E* (−43 ± 1 mV, −55 ± 2 mV, −52 ± 2 mV, and −27 ± 8 mV for basal, ultrasound-stimulated, pressure-stimulated, and heat-stimulated currents, respectively, mean ± SEM, *n* = 3 patches, **P = 0.06, *P = 0.01, one-way ANOVA test with Tukey correction).

While ultrasound stimulation protocols were designed to minimize solution heating, it is conceivable that local temperature of the patched membrane could increase more than bulk solution, perhaps due to absorption and focusing of mechanical energy by the pipette glass. Since TRAAK (30) [and the related K2P channel TREK-1 (37)] are moderately temperature sensitive in the range of 20° to 50 °C in whole-cell recordings [with a threshold of 30°C and maximum estimated Q_10_ ∼6, reviewed in (38)], we performed three control experiments to rule out activation of TRAAK by ultrasonic heating (Fig. 2).

First, we asked whether TRAAK in excised patches is activated by temperature increase. Surprisingly, TRAAK activity is substantially reduced at 40° C relative to 25° C (with a relative activity of 0.25 ± 0.06 at 0 mV at 40° C, mean ± SEM, *n* = 3 patches) (Fig. 2 *A* and *B*). Thermal activation of TREK-1 is also lost upon patch excision (37), but similar inhibition was not observed. The mechanisms underlying patch configuration-dependent effects of temperature on these channels remain to be determined. Second, we asked whether the thermosensitive channel TRPV1 is activated by ultrasound in the same experimental system. While capsaicin robustly activates TRPV1 currents in excised patches (6.5 ± 1.1-fold at +100 mV [mean ± SEM, *n* = 6 patches]), ultrasonic or pressure stimulation had no significant effect on TRPV1 activity (1.0 ± 0.01 and 0.9 ± 0.03-fold activation at +100 mV, respectively [mean ± SEM, *n* = 3 patches]) (Fig. 2 *C* and *D*). Third, we examined the effects of ultrasound stimulation on currents recorded from excised patches containing both TRPV1 and TRAAK channels. Ultrasound and pressure stimulation resulted in hyperpolarizing shifts of the reversal potential toward E_K+_ (from −43 ± 1 mV to −55 ± 2 mV and −52 ± 2 mV, respectively [mean ± SEM, *n* = 3 patches]) (Fig. 2 *E* and *F*), consistent with activation of K^+^-selective TRAAK channels. In contrast, increasing temperature from 25° C to 37° C resulted in a depolarizing shift of the reversal potential (from an average of −43 ± 1 mV to −27 ± 8 mV [mean ± SEM, *n* = 3 patches]) (Fig. 2 *E* and *F*), consistent with thermal activation of cation-nonselective TRPV1 and/or inhibition of TRAAK. We conclude that thermal effects cannot explain ultrasound activation of TRAAK.

**Fig. 3.**
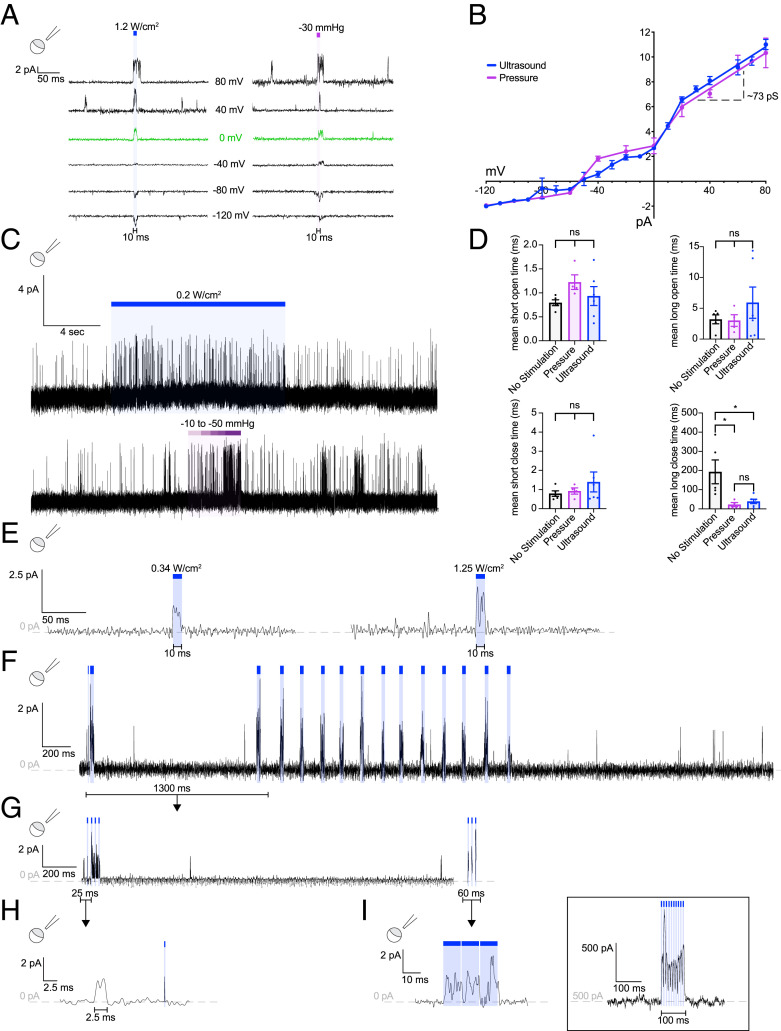
Ultrasound activates single TRAAK channels in *Xenopus* oocyte patches. (*A*) Single TRAAK channel currents recorded at different test voltages (−120 mV (*Bottom*) to +80 mV (*Top*) ΔV = 10 mV, 40 mV increments shown) with 10 ms of stimulation by ultrasound (1.2 W/cm^2^ at 5 MHz, blue bar, *Left*) or pressure (−30 mmHg, purple bar, *Right*). (*B*) The single-channel current elicited by ultrasound (blue) and pressure (purple) versus voltage (*n* = 5 patches). The linear fits from 20 mV to 80 mV and the derived conductance is shown. (*C*) A representative long-duration stimulation by ultrasound (0.2 W/cm^2^ at 5 MHz, blue bar, upper) or pressure (−10 to −50 mmHg, purple bar, lower) (V_test_ = 0 mV). Ultrasound stimulation was performed at low power to minimize bath temperature increases. (*D*) The mean open and closed times of TRAAK channels in the absence of stimulation (*n* = 5 patches from 5 cells), during ultrasound stimulation (0.2 W/cm^2^ at 5 MHz, blue, *n* = 6 patches from 6 cells), and during pressure stimulation (∼−30 mmHg, purple, *n* = 4 patches from 4 cells). Stimulation significantly decreased mean long closed time (*P < 0.01 for pressure or ultrasound versus unstimulated, one-way ANOVA) without significantly changing the mean open or short closed durations. The mean short open times for no stimulation, pressure, and ultrasound were 0.79 ± 0.06, 1.22 ± 0.15, and 0.93 ± 0.20 ms. The mean long open times for no stimulation, pressure, and ultrasound were 3.20 ± 0.72, 3.01 ± 0.94, and 5.9 ± 2.53 ms. The mean short closed times for no stimulation, pressure, and ultrasound were 0.78 ± 0.14, 0.96 ± 0.16, and 1.40 ± 0.52 ms. The mean long closed times for no stimulation, pressure, and ultrasound were 193.10 ± 62.17, 22.61 ± 10.37, and 39.23 ± 11.52 ms. (*E*) The single-channel current response at 0 mV to steps of increasing ultrasound power (0.34 W/cm^2^ and 1.25 W/cm^2^ at 5 MHz, blue bars, V_test_ = 0 mV). (*F*–*I*) A recording demonstrating rapid modulation of channel activity using pulsed ultrasound. (*F*) A 4.5-s record with multiple periods of ultrasound stimulation (blue bars, V_test_ = 0 mV). (*G*) A magnified view of the 1.3-s portion of the record in *F* indicated with a bar. (*H*) A magnified view of 25 ms (indicated in *G*) showing a spontaneous and ultrasound-induced opening (100 µs, 0.34 W/cm^2^, 5 MHz). (*I*) A magnified view of 60 ms (indicated in *G*) showing alternating channel opening and closing in response to pulsed ultrasound (10 ms on, 0.25 ms off, 0.34 W/cm^2^, 5 MHz) (*I*) *Inset*: macroscopic current in *Xenopus* oocyte patch for a comparison to the single channel record (8 ms on, 2 ms off, 0.5 W/cm^2^, 5 MHz).

We performed single-channel recordings of TRAAK to better understand the basis of channel activation. Consistent with macroscopic records, channel activity was low under basal conditions and increased upon pressure or ultrasound stimulation (Fig. 3 *A* and *C*). Single channels were confirmed to be TRAAK by their K^+^ selectivity, single-channel conductance of ∼73 pS at positive potentials, characteristic “flickery” behavior with short openings and a subconductance state, and their absence in patches from control *Xenopus* oocytes (Fig. 3 *A*–*C*). Ultrasound- and pressure-activated channels opened to an indistinguishable full conductance (Fig. 3*B*). In the absence of stimulation, TRAAK had an average open probability of 1.9 ± 0.7%. Ultrasound and pressure increase channel open probability to 6.3 ± 3.3% and 26 ± 9.7%, respectively. The open probabilities reached upon ultrasound and pressure stimulation are not directly comparable because the driving force created by each stimulus was different; relatively high pressures were used to activate channels, but low ultrasound power (0.2 W/cm^2^) was utilized to obtain long (12-s) periods of activation without significant heating (*SI Appendix*, Fig. S2*B*).

**Fig. 4.**
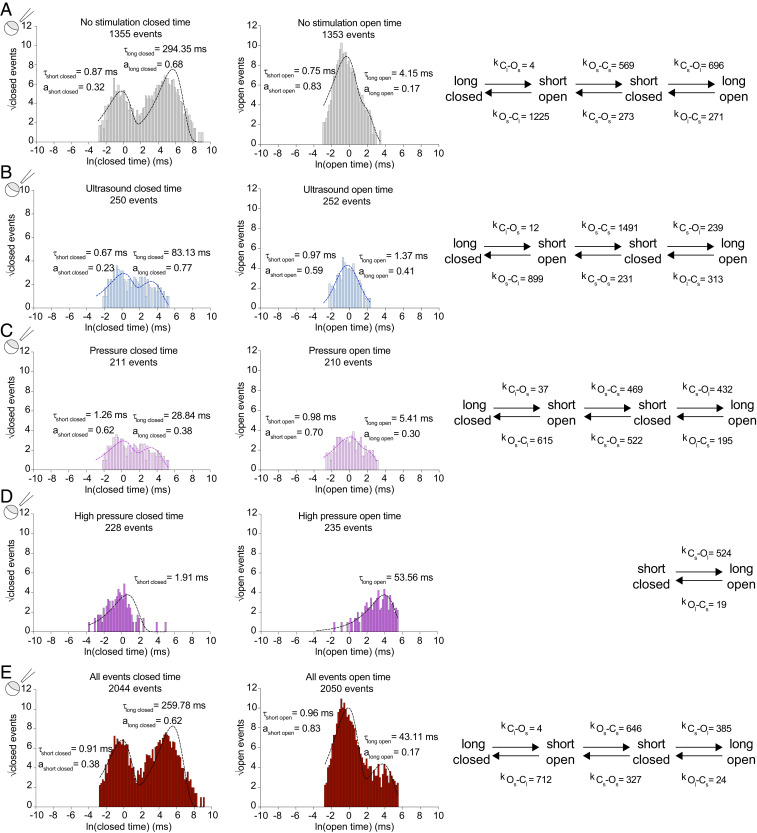
Single-channel analyses and kinetic modeling. (*A*–*E*) Closed and open dwell time histograms from single-channel recordings of TRAAK with corresponding model fit. Representative recordings during (*A*) no stimulation, (*B*) ultrasound stimulation (0.2 W/cm^2^), and (*C*) low pressure (−50 mmHg) stimulation were fit to a four-state (long closed, short open, short closed, and long open) model with eight rates (in s^−1^). A representative recording during (*E*) high-pressure (−150 to −200 mmHg) stimulation was fit to a two-state (short closed and long open) model with two rates. (*E*) Pooled event histograms from all data in *A*–*D* were fit to a four-state model. The closed-time histograms are shown on the left and open-time histograms on the right. The maximum-likelihood fits are shown as dotted lines with mean dwell times (𝜏) and relative proportion (a) of total events shown for each component.

The major effect of either ultrasonic or pressure stimulation is to increase the frequency of channel opening. TRAAK accesses two open states and two closed states distinguished kinetically (Figs. 3*D* and 4). The mean durations of three states, the two open and the short-duration closed (with a dwell time of ∼1 ms), are indistinguishable regardless of whether opening occurs in the absence or presence of stimulation by ultrasound or pressure (Fig. 3*D*). In contrast, the duration of the long-lived closed state is dramatically reduced by either stimulation (from 193.1 ms to 22.6 and 39.2 ms with pressure and ultrasound stimulation, respectively) (Fig. 3*D*). The reduction in long-duration closures explains the increase in open probability upon pressure- or ultrasound-stimulation.

A subtler effect on channel conductance was also observed upon stimulation (Fig. 3*E*). In addition to a full conductance state, TRAAK frequently opens to a subconductance state. Increasing ultrasound power increased the likelihood that a given channel opening would reach full conductance. In the trace shown in Fig. 3*E* recorded at 0 mV, channel openings reached ∼1.25 pA (half conductance) with 0.34 W/cm^2^ ultrasound power and ∼2.5 pA (full conductance) at 1.25 W/cm^2^. Increasing steps of pressure activation similarly increased the likelihood that openings reached full conductance. Together, these results show pressure and ultrasound open TRAAK channels via a shared mechanism that involves destabilizing long-duration closures and favoring full conductance openings with increasing stimulus energy.

We predicted the rapid kinetics of TRAAK activation and ultrasound delivery would permit temporally precise manipulation of single-channel activity. In the record shown in Fig. 3*F*, channels activate upon each of 43 distinct bouts of ultrasound stimulation. Closer inspection shows that even very brief (0.1-ms) periods of stimulation activate TRAAK (Fig. 3 *G* and *H*). TRAAK activity was also modulated using pulsed protocols in which longer periods of stimulation (10 ms) are interleaved with brief periods without stimulation (0.25 ms) (Fig. 3 *G* and *I*). Similar modulation is recorded from patches containing many channels stimulated with pulsed ultrasound in which 8-ms bouts of stimulation are interleaved with 2-ms periods without stimulation (Fig. 3 *I*, *Inset*).

Ultrasound could, in principle, activate TRAAK channels in cell membranes in two fundamentally different ways. First, it could activate the channel directly through the lipid membrane by creating membrane tension that favors channel opening. Alternatively, activation could depend on other factors present in *Xenopus* oocytes or on specific components of the lipid membrane. In that case, energy would be conveyed to the channel in a manner analogous to that proposed for mechanosensitive ion channels that require tethers or second messengers (39, 40). To unequivocally distinguish between these possibilities, we studied channel activation in a fully reduced system. TRAAK was heterologously expressed, purified to homogeneity in detergent to remove all other cellular components, and reconstituted into liposomes of defined lipid composition. The resulting proteoliposomes were blistered and high-resistance patches in the inside-out configuration were formed and recorded under voltage clamp. If ultrasound activates TRAAK in this reduced system, it must work through gating forces conveyed to the channel through the membrane.

**Fig. 5.**
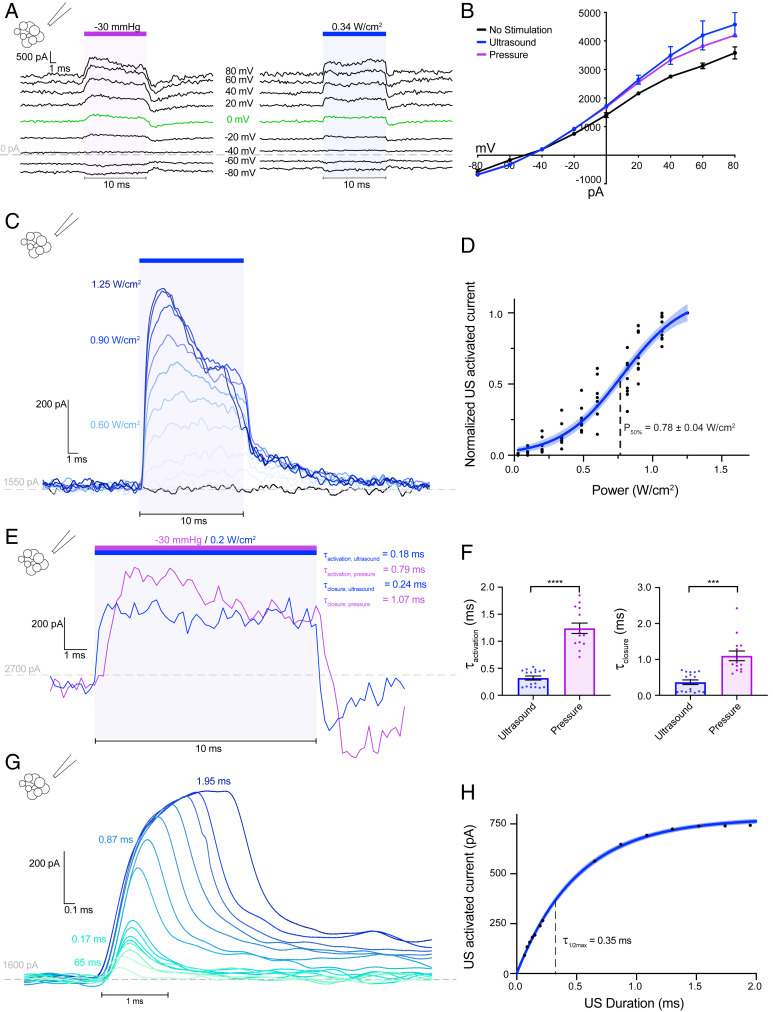
Ultrasound activates purified TRAAK channels reconstituted into lipid membranes. Current recordings from patches of purified TRAAK reconstituted into proteoliposomes. (*A*) Currents recorded during a voltage step protocol (V_hold_ = −50 mV, V_test_ = −80 to +80 mV, ΔV = 10 mV, 20mV increments shown). A pressure (−30 mmHg, purple bar, *Left*) or ultrasound step (0.34 W/cm^2^ at 5 MHz, blue bar, *Right*) was applied during each voltage step. (*B*) The current–voltage relationship of data in *A*. The average current before stimulation (black) and peak currents during pressure (purple) and ultrasound (blue) stimulation are shown (mean ± SEM, *n* = 3 sweeps). (*C*) An overlay of currents during steps of increasing ultrasound power colored from light to dark blue (V_test_ = 0 mV). (*D*) The normalized ultrasound-induced TRAAK current versus ultrasound power (V_test_ = 0 mV). A Boltzmann fit with 95% CI is shown (*n* = 7 patches). (*E*) An overlay of TRAAK current response from the same patch to ultrasound (blue) and pressure (purple) (V_test_ = 0 mV). (*F*) The time constant of channel activation and closure in response to ultrasound and pressure (𝜏_activation,_ _ultrasound_ = 0.32 ± 0.04 ms, 𝜏_activation,_ _pressure_ = 1.24 ± 0.10 ms, 𝜏_close,_ _ultrasound_ = 0.37 ± 0.06 ms, 𝜏_close,_ _pressure_ = 1.10 ± 0.14 ms, *n* = 17 and 14 records for ultrasound and pressure, respectively, from 2 proteoliposomes ****P < 0.0001, Welch’s *t* test). (*G*) An overlay of TRAAK current response to ultrasound stimulation of increasing duration colored from green to blue (V_test_ = 0 mV). (*H*) The maximum current response versus stimulus duration. A fit with 95% CI is shown.

TRAAK currents from proteoliposomes recapitulated channel properties observed in cellular membranes (Fig. 5). Macroscopic currents in the absence of stimulation were K^+^ selective as they reversed near E_K+_ (Fig. 5 *A* and *B*). Pressure steps elicited a rapid increase in current that decayed in the presence of stimulation and rapidly returned to baseline after its removal. As previously reported, the degree of channel activation in this purified system is less than that observed in patches from cells, which is likely due to an increased tension and basal open probability in reconstituted compared to cellular membranes (32). Channels are expected to be oriented randomly in the reconstituted membranes. In both reconstituted and cellular membranes, where channels are oriented uniformly, TRAAK is equivalently activated by positive- and negative-pressure stimulation (32).

Reconstituted TRAAK was robustly activated by ultrasound stimulation (10 ms, 5 MHz, 0.34 W/cm^2^) (Fig. 5 *A* and *B*). Like basal and pressure-activated currents, ultrasound-activated currents were K^+^ selective. Increasing steps of ultrasound power resulted in progressive activation of TRAAK, with a midpoint of ultrasound power activation at 0.78 ± 0.04 W/cm^2^ and 10 to 90% activation observed between 0.2 and 1.25 W/cm^2^ (Fig. 5 *C* and *D*). As observed in patches from *Xenopus* oocytes, ultrasound activation and subsequent channel closure proceeded at faster rates than those observed with pressure (Fig. 5 *E* and *F*, 𝜏_activation,_ _ultrasound_ = 0.32 ± 0.04 ms, 𝜏_activation,_ _pressure_ = 1.24 ± 0.10 ms, 𝜏_close,_ _ultrasound_ = 0.37 ± 0.06 ms, 𝜏_close,_ _pressure_ = 1.10 ± 0.14 ms [mean ± SEM, *n* = 17 records for ultrasound and *n* = 14 records for pressure from 2 proteoliposomes]). Kinetics of channel activation and closing were comparable in patches from proteoliposomes and *Xenopus*; differences in 𝜏_activation,_ _ultrasound_, 𝜏_close,_ _ultrasound_, and 𝜏_close,_ _pressure_ were not statistically significant while a modest difference in 𝜏_activation,_ _pressure_ was (*P* = 0.012, Student’s *t* test). As in patches from *Xenopus* oocytes, a consequence of rapid ultrasound activation is that even brief stimulation can effectively activate channels: ∼50% and ∼95% maximal recruitment is achieved with 0.35 ms and 1.30 ms stimulation, respectively (Fig. 5 *G* and *H*). Recordings from cell membranes and from proteoliposomes show similar power responses and channel kinetics (Figs. 1 *D* and *F* and 5 *D* and *F*), suggesting the same process underlies channel activation in *Xenopus* oocytes and in purified systems. We conclude that ultrasound activation of TRAAK does not require additional cellular components as sensors or conveyors of energy to the channel. Ultrasound activates TRAAK through the lipid membrane.

Could ultrasonic activation of TRAAK in neurons hyperpolarize cells to silence electrical activity? TRAAK is endogenously expressed in myelinated central neurons but is exclusively localized to nodes of Ranvier in mature axons. We reasoned heterologously expressing a soma-targeted version of TRAAK would allow us to address this question with voltage and current clamp experiments in a slice preparation. We therefore generated a TRAAK construct fused to the K_v_2.1 soma-targeting sequence and mRuby for detection (TRAAK-ST-mRuby), in utero electroporated the plasmid into mice, and harvested brain slices from juvenile animals for electrophysiological recordings.

**Fig. 6.**
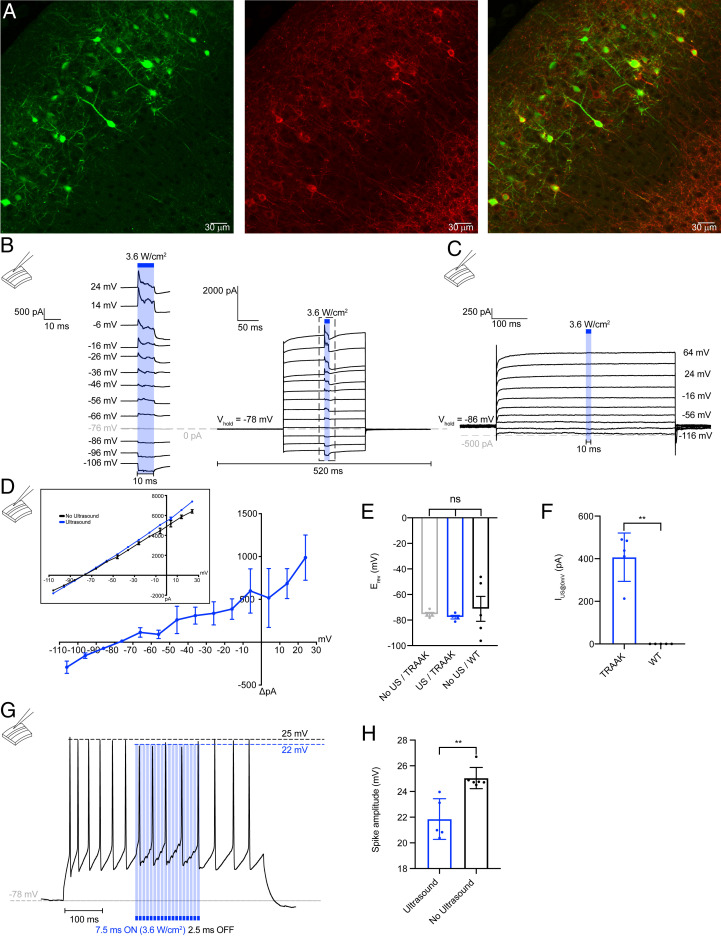
Ultrasound activates TRAAK channels expressed in cortical neurons of mice. (*A*) Representative confocal images of a juvenile mouse cortex in utero electroporated with soluble GFP (green, *Left*) and membrane localized TRAAK-mRuby2 (red, center). The merged image is shown at *Right* (Scale bar, 30 µm). (*B*) Representative whole-cell current recordings from a cortical-layer 2/3 pyramidal-neuron expressing TRAAK during a voltage-step protocol (V_hold_ = −78 mV, V_test_ = −108 to +24 mV). An ultrasound step (3.6 W/cm^2^ at 5 MHz, 10 ms, blue bar) was applied during each voltage step. (*C*) A representative control whole-cell current recordings from a non-TRAAK–expressing cortical layer 2/3 pyramidal neuron during a voltage-step protocol (V_hold_ = −86 mV, V_test_ = −116 to +64 mV). An ultrasound step (3.6 W/cm^2^ at 5 MHz, 10 ms, blue bar) was applied during each voltage step. (*D*) The current–voltage relationship of ultrasound-activated currents from TRAAK-expressing neurons (mean ± SEM, *n* = 3 cells). The average current before and peak current during ultrasound stimulation is plotted in the inset. (*E*) The reversal potential of currents recorded from control (−75.45 ± 1.49 mV, mean ± SEM, *n* = 5 cells) and TRAAK-expressing neurons in the presence (*n* = 4 cells −77.70 ± 1.44 mV) and absence (−71.20 ± 9.74mV, mean ± SEM, *n* = 4 cells) of ultrasound stimulation. (*F*) The peak ultrasound-activated current at 0 mV recorded from TRAAK-expressing neurons (407.40 ± 50.77 pA mean ± SEM, *n* = 5 cells) and negative-control neurons (0 pA, mean ± SEM, *n* = 5 cells **P < 0.01). (*G*) A spike train elicited by the injection of a current (125 pA) into a TRAAK-expressing neuron. Pulsed ultrasound stimulation (7.5 ms on (blue bars), 2.5 ms off, 3.6 W/cm^2^) was applied during the current injection. (*H*) The mean spike amplitude in the presence (21.86 ± 0.71 mV, mean ± SEM, *n* = 5 cells) and absence (25.05 ± 0.33 mV, mean ± SEM, *n* = 6 cells) of ultrasound stimulation (*P* < 0.01).

Confocal images of brain slices showed expression and membrane localization of TRAAK-ST-mRuby channels in cortical-layer 2/3 pyramidal neurons (Fig. 6*A*). Neurons were patched and recorded in whole-cell configuration. Ultrasound stimulation (10 ms, 5 MHz, 3.6 W/cm^2^) activated large currents in TRAAK-ST-mRuby–expressing cells (Fig. 6 *B* and *D*), but not in control (Fig. 6*C*) cells with a mean peak activation of ∼400 pA at 0 mV (Fig. 6*F*). Ultrasound-stimulated currents had rapid opening and closing kinetics (Fig. 6*B*) and reversal near E_K+_ (Fig. 6 *D* and *E*), consistent with activation of TRAAK channels. During a spike train elicited by current injection, pulsed ultrasonic stimulation resulted in phase-matched hyperpolarization during interspike intervals and a ∼3-mV reduction in spike amplitude (Fig. 6 *G* and *H*). We note higher-power ultrasound was required to activate channels in whole-cell recordings from neurons in brain slices compared to excised patches from *Xenopus* oocytes or proteoliposomes. This may be due to differences in recording setup (*SI Appendix*, Fig. S1*B*), patch configuration, or the membrane environment in which TRAAK is embedded. It is also possible that higher power is required because the 400 μm-thick cortical slice absorbs and dissipates some energy from ultrasound stimulation. These results demonstrate that ultrasound can be used to manipulate the activity of TRAAK channels in neurons in the brain. Further optimization of stimulus parameters may be required to maximally activate TRAAK in cells embedded in tissue.

## Discussion

Here, we demonstrate that ultrasound activates mechanosensitive TRAAK ion channels through the lipid membrane in a manner analogous to canonical mechanical activation through increased membrane tension. Ion channels have been increasingly implicated in mediating the cellular electrical effects of ultrasound in excitable cells. The *Escherichia coli* mechanosensitive channel MscL is activated by ultrasound stimulation in the presence of microbubbles to amplify acoustic radiation forces or in the absence of microbubbles using higher-frequency stimulation and a sensitizing mutation (25, 41). Similarly, the *Mus musculus* mechanosensitive channel Piezo1 is activated by ultrasound stimulation in the presence of microbubbles or in their absence using higher-frequency and -power stimulation (22, 42). Behavioral responses of *C. elegans* to ultrasound require the mechanosensitive MEC-4 channel (20). While *C. elegans* TRP-4 had initially been genetically implicated in behavioral responses to ultrasound using a *trp-4(ok1605)* strain (43), the involvement of TRP-4 in ultrasound response was later ruled out using additional *trp-4* mutant strains and the initial results ascribed to mutation(s) in the genetic background of *trp-4(ok1605)* nematodes (20). Noncanonically mechanosensitive channels have also been implicated in cellular responses to ultrasound. Astrocytic TRPA1 accounts for some behavioral and cellular responses to low-frequency, low-intensity ultrasound (0.4 MHz, 0.3 W/cm^2^) in mice (23). Voltage-gated channels, some of which are mechanically sensitive, have been implicated in neural responses to ultrasound (24, 44), although other studies report modest effects that may be explained by ultrasound-induced temperature changes (21, 22). Other tension-gated mechanosensitive channels could be similarly ultrasound-sensitive (39).

These results contrast with a previous report of ultrasound activation of TRAAK and related TREK channels in oocytes (21). In the current study, brief duration and low power stimulation (1.2 W/cm^2^, 5 MHz, 10 ms) robustly activates TRAAK (up to ∼20-fold) with very fast kinetics (𝜏_activation_, _ultrasound_ ∼250 μs). Ultrasound activation of TRAAK reported here closely corresponds to canonical–mechanical activation in whole cells, patches from *Xenopus* oocytes or proteoliposomes, and single-channel recordings. In previous work, long-duration and higher-power stimulation (2 W/cm^2^, 5 MHz, 1 s) modestly activated channels (up to ∼0.15-fold) with 1,000-fold slower kinetics (𝜏 ∼800 ms) (21).

What is the physical basis for ultrasound activation of TRAAK? Temperature increase, cavitation, displacement, and acoustic scattering are unlikely to account for TRAAK activation for the following reasons. The stimulation protocols used here are expected to result in minimal heating, consistent with previous reports (20, 21, 23, 43). TRAAK and related TREK channel thermosensitivity have been reported to require cell integrity (30, 37) and we show (Fig. 2 *A* and *B*) that TRAAK activity is in fact reduced by ∼75% at 40 °C relative to 25 °C in the excised patch configuration used in experiments here (Figs. 1 and 3–5) (31). We further demonstrate, using the same experimental setup, that thermosensitive TRPV1 channels are not activated by ultrasonic stimulation that activates TRAAK. Temperature increase can therefore not explain TRAAK activation we observe in patch recordings. Cavitation requires approximately 10-fold higher acoustic pressures than achieved here and would open nonselective, rather than K^+^-selective, holes in the membrane. The expected displacement gradient is too small (∼0.1 µm) over the ∼300-µm ultrasound wavelength to account for the activation we observe. Similarly, scattering by the glass pipettes is unlikely to substantially change the ultrasound intensity profile since the tip diameter is small (1 µm) relative to ultrasound wavelength.

We conclude that energy from ultrasound most likely increases membrane tension to promote TRAAK channel opening. Acoustic radiation forces and resulting acoustic streaming can account for this mechanical consequence of ultrasound stimulation (5, 12, 22). This best explains the common basis for TRAAK activation by ultrasound or pressure stimulation. Direct comparison of membrane tension during pressure and ultrasound stimulation (for example, by imaging patch geometry during channel activation and calculating tension) would further support this conclusion and could provide insight into protocols that maximally increase tension and optimally activate channels. Ultrasound activation of other mechanosensitive channels might involve other mechanical processes in addition to the generation of membrane tension. For example, mechanical lipid demixing activates phospholipase D2 which, in turn, activates TREK1 channels through the generation of the signaling-lipid phosphatidic acid (45).

Ultrasound has both suppressive and stimulatory effects on neuronal activity, depending on the stimulus design and tissue under study. Inhibitory effects of ultrasound have been demonstrated in the central and peripheral nervous systems including in studies of light-evoked potentials in the visual cortex, pupillary reflexes, spreading cortical depression, and sciatic nerve activity. The underlying molecular mechanisms for these effects are unknown. Our results suggest that TRAAK- and TREK-mechanosensitive K^+^ channels are responsible for some ultrasound-induced inhibition of neuronal activity. TRAAK and TREK1 channels are localized to nodes of Ranvier within myelinated axons, and their activation is expected to impact spiking by increasing resting K^+^ conductance and hyperpolarizing cells (33, 34). Focused ultrasound stimulation of myelinated fibers containing TRAAK and TREK may be a viable strategy for targeted suppression of neural activity.

An alternative to manipulating endogenously expressed channels is to sensitize targeted cells with overexpression of an ultrasound-activated protein. Several such “sonogenetic” approaches have been reported using MscL and Piezo ion channels as ultrasound actuators for neuromodulation or expression of reporter genes (43, 25, 41, 42, 46). Our results provide a framework for the development of TRAAK or other mechanosensitive ion channels as modular tools for targeted suppression or activation of electrically excitable cells. The low resting open probability and relatively high conductance [compared to channel rhodopsins used for optogenetics (47)] of TRAAK make it a promising target for further engineering to optimize expression, subcellular localization, and ultrasound-responsive range for sonogenetic applications in tissue.

## Methods

### Expression in and Recording from *Xenopus Laevis* Oocytes.

A construct encoding full-length *Homo sapiens* TRAAK (UniProt Q9NYG8-1) was codon optimized to enhance eukaryotic expression in *P. pastoris*, *Spodoptera frugiperda*, and *H. sapiens* (without changing the native amino acid sequence), synthesized (Genewiz), and cloned into a modified pGEMHE vector using Xho1 and EcoR1 restriction sites. The transcribed message encodes *H. sapiens* TRAAK amino acids 1 to 393 with an additional amino acid sequence of “SNS” at the C terminus. A construct encoding *M. musculus* TASK2 (UniProt Q9JK62-1) was codon optimized to enhance eukaryotic expression in *P. pastoris*, *S. frugiperda*, and *H. sapiens* (without changing the native amino acid sequence), synthesized (Genewiz), and cloned into a modified pGEMHE vector using Xho1 and EcoR1 restriction sites. The region encoding the C terminus was truncated such that the transcribed message encodes *M. musculus* TASK2 amino acids 1 to 335 with an additional amino acid sequence of “SNS” at the C terminus. The impact of codon optimization on protein expression was not evaluated in any expression system. A construct encoding *M. musculus* TRPV1 (UniProt Q704Y3) was cloned into a modified pGEMHE vector using PCR and Gibson assembly and an endogenous NheI cut site at codon 5 was removed by Quickchange PCR. The transcribed message encodes full-length TRPV1 with an additional amino acid sequence of “SNS” at the C terminus.

Complementary DNA (cDNA) was transcribed from these plasmids in vitro using T7 polymerase and 50 nl containing 0.1 to 10 ng complimentary RNA (cRNA) was injected into *Xenopus laevis* oocytes extracted from anesthetized frogs. Currents were recorded at 25 °C from inside-out patches excised from oocytes 1 to 5 d after cRNA injection. For TRAAK-expressing and TRAAK- and TRPV1-coexpressing oocyte patch recordings, the pipette solution contained the following: 14 mM KCl, 126 mM NaCl, 2 mM MgCl_2_, 10 mM HEPES, pH = 7.4 with KOH and the solution in the bath and ultrasound chamber contained 140 mM KCl, 2 mM MgCl_2_, 10 mM HEPES, 1 mM EGTA, pH = 7.1 with KOH. For TRPV1 oocyte recordings, both the pipette and bath solutions contained the following: 140 mM NaCl, 2 mM MgCl_2_, 10 mM Hepes, pH = 7.1 with NaOH. Currents were recorded using an Axopatch 200B Patch Clamp amplifier at a bandwidth of 1 kHz and digitized with an Axon Digidata 1550B at 500 kHz. Pressure was applied with a High-Speed Pressure Clamp device (ALA Scientific Instruments). Single-channel patches were identified as long (minimum 3 min) recordings without superimposed channel openings after pressure-induced increase in open probability. To evaluate channel open and close durations, currents from patches containing no superimposed channel openings were unfiltered in order to preserve very brief openings that are characteristic of TRAAK. Single-channel open events were idealized by half-amplitude threshold crossing. All single-channel data were analyzed using custom written software (48).

### TRAAK Reconstitution and Recording in Proteoliposomes.

Mouse TRAAK (UniProt O88454-1) was cloned and expressed in *Pichia pastoris* cells as previously described (49) with modifications described here. The construct used for purification included an additional 26-amino acid N-terminal sequence from Q9NYG8-1 that improved heterologous expression. The final construct is C-terminally truncated by 97 amino acids, incorporates two mutations to remove *N*-linked glycosylation sites (N81Q/N84Q), and is expressed as a C-terminal PreScission protease-cleavable EGFP-10xHis fusion protein. As a result, there is an additional amino acid sequence of “SNSLEVLFQ” at the C terminus of the final purified protein after protease cleavage.

Frozen *Pichia* cells expressing TRAAK were disrupted by milling (Retsch model MM301) five times for 3 min at 25 Hz. All subsequent purification steps were carried out at 4 °C. Milled cells were resuspended in buffer A (50 mM Tris pH 8.0, 150 mM KCl, 1 mM EDTA, 0.1 mg/mL DNase1, 1 mg/mL pepstatin, 1 mg/mL leupeptin, 1 mg/mL aprotinin, 10 mg/mL soy trypsin inhibitor, 1 mM benzamidine, 100 µM AEBSF, 1 µM E-64, and 1 mM phenylmethysulfonyl fluoride added immediately before use) at a ratio of 1 g of cell pellet per 4 mL of lysis buffer and sonicated for 4 min with a 25% duty cycle. The solution was ultracentrifuged at 150,000 xg for 1 h at 4 °C. Pellets were transferred to a Dounce homogenizer in buffer B (buffer A + 1% n-dodecyl ß-D-matoside (DDM)/0.2% cholesterol hemisuccinate (CHS). Detergent was added from a 10% DDM/2% CHS stock in 200 mM Tris pH 8 that was sonicated until clear. Following homogenization, solutions were stirred for 2 h at 4 °C followed by centrifugation at 35,000 g for 45 min. Anti-Green Fluorescent Protein (GFP) nanobody resin washed in buffer B was added to the supernatant at a ratio of 1 mL resin:1 mg purified anti-GFP nanobody/15 g *Pichia* cells and stirred gently for 2 h. Resin was collected on a column and serially washed in buffer C (buffer A + 0.1% DDM/0.02% CHS), buffer D (buffer A + 150 mM KCl + 0.1% DDM/0.02% CHS). The resin was resuspended in two volumes of buffer C with 1 mg purified Precission protease and gently rocked in column overnight. Cleaved TRAAK was eluted in ∼4-column volumes of buffer C, spin concentrated (50 kDa molecular weight cut off (MWCO), and applied to a Superdex 200 column (GE Healthcare) equilibrated in buffer E (20 mM Tris pH 8.0, 150 mM KCl, 1 mM ethylenediaminetetraacetic acid (EDTA), 0.025%/0.005% DDM/CHS). Peak fractions were pooled and concentrated to ∼1 mg/mL for reconstitution.

Purified TRAAK was reconstituted in l-α-phosphatidylcholine extract from soybean lipids as described (50). Proteoliposomes were snap-frozen in liquid nitrogen and stored at −80 °C in De/Rehydration (DR) buffer composed of 200 mM KCl, 5 mM Hepes-KOH pH to 7.2. When preparing proteoliposomes for patching, samples were thawed at room temperature and dried for 2.5 to 3 h in a vacuum chamber to dehydrate. The dehydrated proteoliposomes were then rehydrated with 20 µL DR buffer. Currents were recorded at 25 °C from inside-out patches excised from proteoliposomes for at least 12 h after rehydration. Pipette solution contained the following: 5 mM Hepes, 20 mM KCl, 180 mM NaCl, pH 7.2 adjusted with NaOH. Bath solution contained the following: 5 mM Hepes, 200 mM KCl, 40 mM MgCl_2_, pH 7.2 adjusted with KOH. Currents were recorded using an Axopatch 200B Patch Clamp amplifier at a bandwidth of 1 kHz and digitized with an Axon Digidata 1550B at 500 kHz.

### Ultrasound Setup and Application.

Inside-out patches excised from either oocytes or proteoliposomes were quickly (within 5 to 10 s) transferred to the ultrasound chamber. The patch was centrally positioned ∼1 in (25.4 mm) over the cylindrical transducer base surface, separated only by bath solution. The connection between the base of the ultrasound transducer and bath was made using a clear, nontoxic polyvinyl chloride (PVC) tubing which fit over the outer diameter of the transducer. An ultrasound wave was generated using a V326-SU (Olympus) focused-immersion ultrasonic transducer with a 0.375 in (9.525 mm) nominal element diameter, which had a focal point at 25.2 mm (0.993 in), and an output center frequency of 4.78 MHz. A function generator (Agilent Technologies, model 33220A) was used to trigger the transducer’s ultrasound pulses. For *Xenopus laevis* oocytes and proteoliposome patches, an ENI RF (radio frequency) amplifier (model 403LA) was used. For whole-cell brain slice electrophysiology, an Amplifier Research 5S1G4 RF amplifier was used. Both RF amplifiers received an input voltage waveform from the function generator and provided the output power to the ultrasound transducer for producing the acoustic pressure profile of a stimulus waveform. The timing of the ultrasound stimuli was controlled by triggering the function generator manually or by software (Clampex 10.7). In the case of ultrasound pulse generation though software, a Clampex 10.7–generated waveform triggered a first function generator through a digitizer (Axon Digidata 1550B), which triggered a second function generator, which triggered the RF amplifier that drives the ultrasound transducer. Solutions were degassed to minimize microbubble cavitation and ultrasound attenuation.

### In Utero Electroporation.

Electroporation was performed on pregnant CD1 (ICR) mice (E15, Charles River ca. SC:022) as described (51). For each surgery, the mouse was initially anesthetized with 5% isoflurane and maintained with 2.5% isoflurane. The surgery was conducted on a heating pad, and warm, sterile phosphate buffered saline was intermittently perfused over the pups throughout the procedure. A micropipette was used to inject ∼1 μL of recombinant DNA at a concentration of 2 μg/μL and into the left ventricle of each embryo’s brain (typically DNA encoding TRAAK was doped with plasmid expressing GFP at a concentration of 1:3 to facilitate screening for expression after birth). Fast-green (Sigma-Aldrich) was used to visualize a successful injection. Following successful injection, platinum-plated forceps-type electrodes (5-mM Tweezertrodes BTX Harvard Apparatus) connected to the negative pole were used to gently grab both sides of the embryo’s head and the third electrode connected to the positive pole was placed slightly below lambda (52). An Electro Square Porator (BTX Harvard Apparatus) was used to administer a train of 6 × 40 mV pulses with a 1-s delay. After the procedure, the mouse was allowed to recover and come to term, and the delivered pups were allowed to develop normally. On the day of birth, animals were screened for location and strength of electroporation by transcranial epifluorescence under an Olympus MVX10 fluorescence stereoscope. The sex of animals used for slice electrophysiology or microscopy was not determined.

### Slice Electrophysiology.

We used radial slices from the somatosensory barrel cortex cut along the thalamocortical plane or coronal-cortical sections. The hemisphere was trimmed on both the anterior and posterior side of the barrel cortex with coronal cuts, placed on its anterior side, and a cut was made with a scalpel so that much of barrel cortex lay in a plane parallel to the cut. The surface of this last cut was glued to the slicer tray. The preparation was aided by the use of epifluorescent goggles to visualize the expressing area. Six 300-μm slices were prepared. Cortical slices (400 μm thick) were prepared as described (53) from the transfected hemispheres of both male and female mice aged P15 to P40 using a DSK Microslicer in a reduced sodium solution containing the following: 83 mM NaCl, 2.5 mM KCl, 3.3 mM MgSO_4_, 1 mM NaH_2_PO_4_, 22 mM glucose, 72 mM sucrose, and 0.5 mM CaCl_2_ and stored submerged at 34 °C for 30 min, then at room temperature for 1 to 4 h in the same solution before being transferred to a submerged recording chamber maintained at 25 °C in a solution containing the following: 119 mM NaCl, 2.5 mM KCl, 1.3 mM MgSO_4_, 1.3 mM NaH_2_PO_4_, 20 mM glucose, 26 mM NaHCO_3_, and 2.5 mM CaCl_2_. Pipettes were filled with potassium-gluconate–based internal solution containing the following: 135 mM K-gluconate, 8 mM NaCl, 10 mM Hepes, 0.3 mM Na_3_GTP, 4 mM MgATP, and 0.3 mM EGTA. Currents were recorded using an Axopatch 200B Patch Clamp amplifier at a bandwidth of 1 kHz and digitized at 500 kHz. The recording chamber contained a cortical slice resting over a thin film of mylar. Directly under the mylar was a 1-inch, nontoxic PVC tube leading to the surface plane of the ultrasound transducer.

### Ultrasound-Induced Temperature Changes.

Temperature changes generated by stimulation with the 5 MHz immersion focus ultrasonic transducer (V326-SU, Olympus) were measured over time with an immersed thermocouple at the position of maximum ultrasound power. The function generator was set to 1.0 V, 5 MHz sine wave, and infinite cycles. These measurements corresponded well to expected temperature increases calculated with the following relationships (54):

$$\Delta T=\frac{Q\Delta t}{C\varrho },$$ [1]

$$Q=\frac{\alpha P^{2}}{\varrho c},$$ [2]

where *ΔT* is temperature change, *Q* is ultrasound-generated heat, *Δt* is time of ultrasound stimulation in seconds, *C* is solution specific heat capacity (3,600 J kg^−1^ ⋅ K^−1^), ϱ is solution density (1,028 kg ⋅ m^−3^), α is ultrasound absorption coefficient (20 m^−1^), *P* is effective ultrasound pressure (calculated as 0.707 multiplied by the peak amplitude of the sine wave), and c is the speed of sound in solution (1,515 m ⋅ s^−1^). These relationships were subsequently used to estimate temperature increases and design protocols that minimized heating.

### Calculating Ultrasound Pressure and Power Intensity.

The output pressures were measured using a calibrated hydrophone (Onda, model HNR-0500). The hydrophone measurements were performed at the position of peak spatial pressure. When converting the measured voltages into pressures, we accounted for the hydrophone capacitance according to the manufacturer’s calibration. Using the appropriate conversion factor listed under the Pascals-per-volt column on the look-up table that was supplied with the calibrated hydrophone, the hydrophone voltage-trace waveform was transformed into an acoustic-pressure waveform measured in MPa.

We calculated the ultrasound power intensity in Watts/square centimeter (W/cm^2^) with the following equation:

$$I=\frac{P^{2}}{Z}=\frac{{\left(P\times 0.707\right)}^{2}}{\left(1.48\times 10^{6}\frac{kg}{m^{2}.s}\right)}\left(\frac{1}{100^{2}}\right),$$ [3]

where *P* is effective ultrasound pressure (calculated as 0.707 multiplied by peak amplitude of the pressure wave) and *Z* is the acoustic impedance (1.48 × 10^6^ kg ⋅ m^−2^ ⋅ s^−1^ was used).

### Animals.

Animal procedures were reviewed and approved by the Animal Care and Use Committee at the University of California, Berkeley (AUP 2016-09-9174, AUP 2014010-6832, and AUP-2015-04-7522-1).

## Supplementary Material

Supplementary File

## Data Availability

All study data are included in the article and/or *SI Appendix*.
