# Supplementary material for: Ultrasound activates mechanosensitive TRAAK K+ channels through the lipid membrane
Source: Proc Natl Acad Sci U S A. 2021 Feb 4;118(6):e2006980118. doi: 10.1073/pnas.2006980118 (PMC8017979; doi:10.1073/pnas.2006980118)
Supplement: Supplementary File [file pnas.2006980118.sapp.pdf]

PNAS
www.pnas.org

Supplementary Information for

Ultrasound activates mechanosensitive TRAAK K+ channels through the lipid membrane

Ben Sorum[1,2], Robert A. Rietmeijer[1,2,3], Karthika Gopakumar[1,2], Hillel Adesnik[1,2] & Stephen G. Brohawn[1,2*]

Affiliations

[1] Department of Molecular & Cell Biology, University of California Berkeley, Berkeley, California 94720, USA
[2] Helen Wills Neuroscience Institute, University of California Berkeley, Berkeley, California 94720, USA
[3] Biophysics Graduate Program, University of California Berkeley, Berkeley, California 94720, USA

Stephen G. Brohawn
Email: brohawn@berkeley.edu

This PDF file includes:

Figures S1 to S2

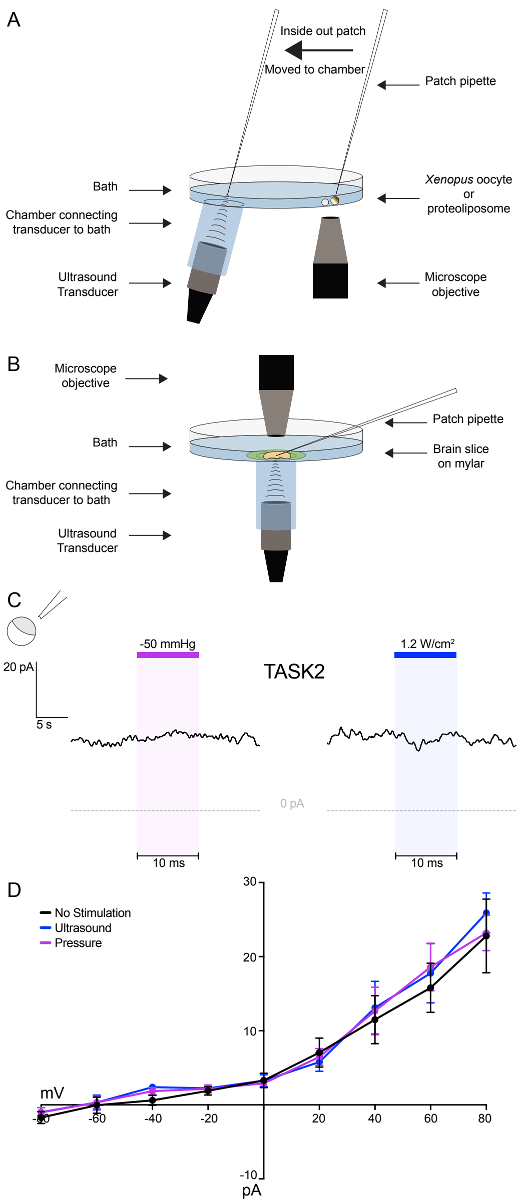

Fig. S1. Setup for recording ultrasound effects on ion channels

(A) Chamber design for patch recording experiments. An ultrasound transducer is connected to the bath solution through PVC tubing ~1 inch in height (25.4 mm) which fit over the outer diameter of the transducer such that an unobstructed liquid column exists between the transducer face and patch pipette. (B) Chamber design for slice recording experiments. An ultrasound transducer is connected to the bath solution through PVC tubing ~1 inch in height (25.4 mm) which fit over the outer diameter of the transducer such that a thin mylar sheet rests over the PVC tubing on which the brain slice is fixed. (C) Voltage-clamp recording from an inside-out patch containing the non-mechanosensitive K2P channel TASK2 (V_{test} = 0 mV). Neither pressure nor ultrasound activates TASK2 currents. (D) Current-voltage relationship from TASK2-containing patches. Average current in the absence of stimulation (black) and maximum currents during pressure (purple) and ultrasound (blue) stimulation are shown (mean ± SEM, n = 3 cells). Recordings were made in a 10-fold $[K^+]$ gradient and presented in physiological convention: E_{K+} = -59 mV and positive current indicates K^+ flow from the high $[K^+]$ (intracellular) to low $[K^+]$ (extracellular) side.

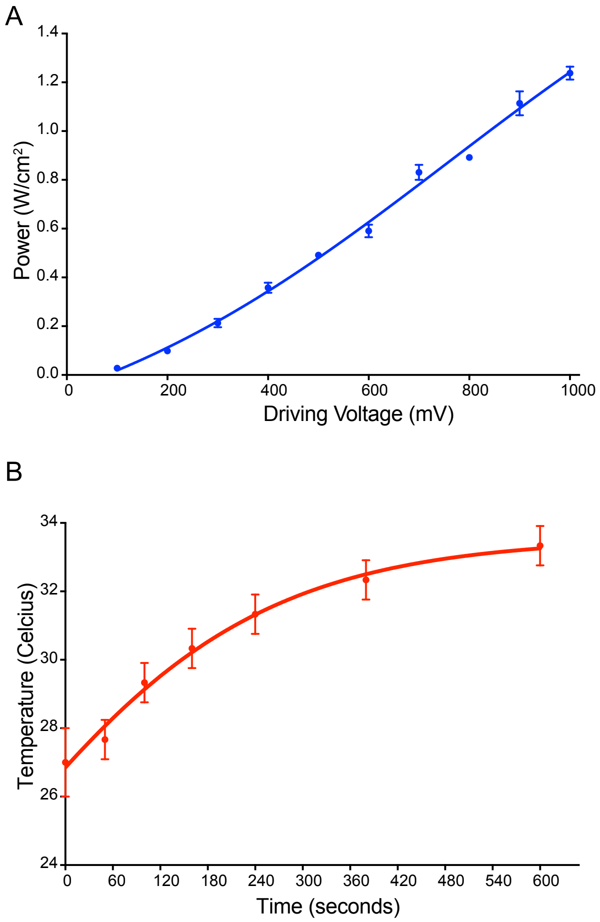

Fig. S2. Calibration of ultrasound power and temperature increases
(A) Power versus driving voltage. Pressure was measured at the point of maximum ultrasound intensity using a manufacturer-calibrated needle hydrophone and converted to power as described in Methods. (B) Temperature versus ultrasound stimulation time. Temperature increases at the point of maximum ultrasound intensity were measured using a thermocouple during constant stimulation at 1.2 W/cm^2.
